# Non-Medical Gender Affirming Practices Among Transgender Individuals: A Systematic Review on the Health Implications of Chest Binding and Genital Tucking

**DOI:** 10.1080/19317611.2025.2560416

**Published:** 2025-09-15

**Authors:** Thanapob Bumphenkiatikul, Theeradon Sakpetch, Pakin Sungnuch, Xi Huang, Somboon Hataiyusuk, Sorawit Wainipitapong

**Affiliations:** ^a^ Center of Excellence in Transgender Health, Faculty of Medicine, Chulalongkorn University, Bangkok, Thailand; ^b^ Division of Academic Affairs, Faculty of Medicine, Chulalongkorn University, Bangkok, Thailand; ^c^ Faculty of Medicine, Chulalongkorn University, Bangkok, Thailand; ^d^ Department of Educational Psychology, Faculty of Education, Chinese University of Hong Kong, Hong Kong; ^e^ Department of Psychiatry, Faculty of Medicine Siriraj Hospital, Mahidol University, Bangkok, Thailand; ^f^ Health Service and Population Research Department, Institute of Psychiatry, Psychology and Neuroscience, King’s College London, London, UK; ^g^ Department of Psychiatry, Faculty of Medicine, Chulalongkorn University and King Chulalongkorn Memorial Hospital, Bangkok, Thailand; ^h^ Department of Global Health and Social Medicine, King’s College London, London, UK

**Keywords:** Chest binding, genital tucking, transgender, gender dysphoria, gender incongruence

## Abstract

**Objectives:**

This systematic review describes health implications of two non-medical gender affirming practices: chest binding and genital tucking.

**Methods:**

In January 2024, three databases (MEDLINE, Embase, and APA PsycINFO) were systematically searched using two comprehensive search terms related to: (1) chest binding or genital tucking, and (2) transgender people. Publications not in English, non-peer reviewed, insufficient data, and any forms of reviews were excluded. A manual search of relevant reviews and references was additionally done for additional resources. An updated search was done in July 2024. This systematic review was registered with PROSPERO (CRD42024500400) and followed the Preferred Reporting Items for Systematic Reviews and Meta-Analyses (PRISMA) guidelines.

**Results:**

This review included 18 studies (11 on chest binding, 7 on genital tucking) with a total of 3,235 participants. Negative implications were frequently reported, including dermatological issues, pain, hyperprolactinemia, breathing difficulties, testicular torsion, and poorer semen quality. However, some studies also found positive effects on dysphoria, life satisfaction, and mental health. Several studies highlighted lack of knowledge among healthcare providers about these practices.

**Conclusion:**

Chest binding and genital tucking have complicated tradeoffs between physical health risks and potential psychological benefits of reduced dysphoria. Further research is needed, especially on long-term effects, safer methods, and promoting education to build a supportive healthcare environment.

## Introduction

Gender affirmation has been evidenced to promote mental health and satisfaction among some transgender individuals (Harris, 2023), either by using hormonal or surgical affirmation (Nimitpanya et al., 2022; Park et al., 2022; Pliensak et al., 2023). However, beyond these medical approaches of affirmation, there exist non-medical methods such as chest binding and genital tucking that serve diverse functions across varied contexts. These practices are commonly adopted by a significant number of transgender individuals at different stages of gender exploration and transition (Peitzmeier et al., 2017). Before accessing medical gender affirmation, many transgender individuals use these practices as initial steps in their transition process (Wainipitapong et al., 2022). However, such practices are also employed by non-transgender individuals such as drag performers and gender artists, non-binary individuals managing fluctuating dysphoria, cross-dressers, and others experimenting with gender expression, each with distinct motivations, techniques, and purposes or meanings.

Chest binding is an act of compressing chest tissues to achieve a flattened appearance, aiming to alleviate distress related to chest dysphoria, especially in individuals assigned female at birth with gender dysphoria (Julian et al., 2021). It encompasses various methods and materials, including purpose-made binders, sports bras, compression shirts, and different types of tape or bandages. It is particularly important for gender-diverse people who do not have access to mastectomy. Despite its advantages for affirmation purposes, chest binding has been associated with several negative consequences, including itches, pain, and overheating (Peitzmeier et al., 2017). One scoping review suggests that chest binding generally has negative physical health effects, especially for those with larger chests, yet many continue to bind due to its identity and mental health benefits (Pehlivanidis & Anderson, 2025).

Conversely, among transgender individuals assigned male at birth, genital tucking (moving testes up into the inguinal canals and positioning the penis and scrotum between the buttocks) is commonly practiced, contributing to a more feminine appearance (Malik et al., 2024). Genital tucking techniques vary and may involve specialized undergarments (gaffs), medical or athletic tape, or other compression methods. Both genital tucking and chest binding can also be practiced independently of gender affirmation purposes and may be used by individuals without any desire for medical gender affirmation. The duration and frequency of use for both practices varies considerably among individuals, ranging from occasional use for specific social situations to daily, prolonged wear. However, there is evidence of a link between tucking and reduced fertility (de Nie et al., 2022). Only half of the transgender individuals practicing tucking reported concern about health effects, which commonly include itches, rash, and testicular pain (Malik et al., 2024).

The use of these practices varies significantly across cultural contexts - from urban transgender communities with established peer networks and resources, to rural settings with limited community support, to different cultural frameworks that may conceptualize gender and bodily modification differently. Usage patterns also differ across phases of gender journey: initial exploration and experimentation, daily management during social transition, temporary use for specific social or professional contexts, methods to alleviate body dysphoria, or as interim measures while accessing medical care. The social and cultural strategies surrounding these practices - including community knowledge sharing, ritualistic aspects, peer mentoring, and integration with other gender-affirming activities - remain largely understudied.

With the increasing numbers of transgender individuals, the evaluation of both advantages and disadvantages associated with these practices holds significant importance for this group and their healthcare practitioners, particularly in regions with limited access to medical gender affirmation services. In addition, there is a notable absence of comprehensive systematic reviews addressing these non-medical affirming practices. Although these practices may be used sporadically by cross-dressers and drag performers, our review targets transgender populations where chest binding and genital tucking are typically sustained over longer periods, warranting systematic examination of their health implications. Therefore, accordingly, the objective of our review is to provide a detailed descriptive analysis elucidating the positive and negative implications of chest binding and genital tucking among transgender individuals. Insights from our review will guide education for both transgender populations and healthcare professionals regarding the benefits and potential adverse effects of these practices, informing decisions about timing, identifying serious risks, and highlighting the need for preparatory considerations such as fertility preservation.

## Methods

### Search strategy

Our review was registered with PROSPERO (CRD42024500400) and followed the Preferred Reporting Items for Systematic Reviews and Meta-Analyses (PRISMA) guidelines (Moher et al., 2009). In January 2024, three databases (MEDLINE, Embase, and APA PsycINFO) were systematically searched using two comprehensive search terms related to 1) chest binding or genital tucking, and 2) transgender people, with no restrictions on publication date. An updated search was conducted for additional publications in July 2024. We excluded publications not in English, non-peer reviewed, those with insufficient data (e.g., poster or abstract), and any forms of reviews. A manual search of relevant reviews and references was additionally done for additional resources. Given the limited research on these practices and their historical documentation mainly through case reports, we included case reports and case series in our review. This review required no ethical approval. The search terms and eligibility criteria are shown in Table 1.

**Table 1.** Search strategy and eligibility criteria.

Parameters	Inclusion		Exclusion
Article profiles	Any methodological designs will be considered for inclusion as appropriate. No restrictions on the publication date		Non-peer reviewed Reviews, systematic reviews, meta-analyses No full text is available Not published in English Insufficient data
Populations	Transgender individuals practicing chest binding and/or genital tucking for gender affirmation purposes		Individuals practicing chest binding and/or genital tucking for non-gender affirmation purposes
Intervention	Chest binding Genital tucking		–
Control	Transgender individuals not practicing chest binding and/or genital tucking		–
Outcomes	Physical and mental implications		–
Search terms			
chest binding or genital tucking		(chest binding or chest flattening or breast binding or tucking).mp.	
transgender people		For APA PsycINFO – lgbtq [MeSH]; For MEDLINE and Embase – “sex and gender minority” [MeSH] OR (transgender* or gender dysphori* or gender incongruent* or transsex* or trans*man or trans*men or trans*ine).mp.	

### Study selection

Deduplication was done by SW. Title and abstract screening was independently conducted by two reviewers (SW and SH) using Covidence (Covidence systematic review Software, Veritas Health Innovation, Melbourne, Australia). Full-text reviews of selected articles were then conducted independently by SW and SH. SW extracted data from the studies included and rated their methodological qualities using the Joanna Briggs Institute (JBI) instruments (Barker et al., 2023).

### Quality appraisal

The JBI instruments are critical appraisal tools, including specific checklists for various methodological designs, such as qualitative studies, analytic cross-sectional studies, case-control studies, and case reports (Munn et al., 2023). These tools align with the methodological designs of the studies included in our review. These checklists comprise up to ten items, based on methodological designs, measuring essential components of methodological qualities by responding ‘Yes’, ‘No’, ‘Unclear’, and ‘Not applicable’.

### Data analysis

Data from included studies were integrated in a narrative synthesis according to examined outcomes. Statistical values such as count, percentage, mean, and standard deviation were extracted or calculated and tabulated in the result section.

## Results

**Figure 1.**
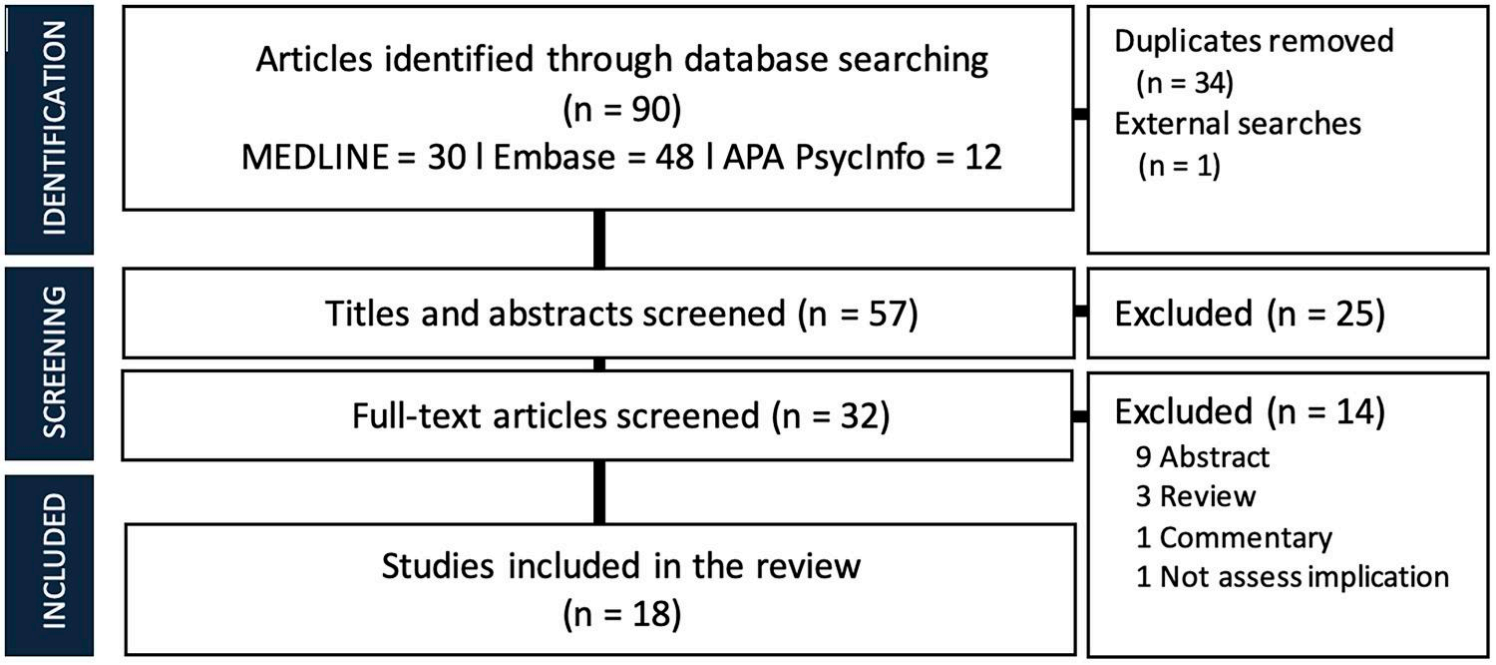
The Preferred Reporting Items for Systematic Reviews and Meta-Analyses (PRISMA) flowchart.

From three databases and external searches, we initially identified a total of 73 articles and ultimately included 18 studies in our review (see Figure 1) (de Nie et al., 2022; Debarbo, 2020; Finney et al., 2024; Jarrett et al., 2018; Julian et al., 2021; Kidd et al., 2024; Kim et al., 2022; Lee et al., 2019; Malik et al., 2024; Patel & Abramowitz, 2020; Pehlivanidis & Anderson, 2024; Peitzmeier et al., 2017, 2021; Reddy-Best et al., 2023; Schultz et al., 2021; Subedi et al., 2025; Trussler & Carrasquillo, 2020; Turley & Potdar, 2023), with 11 papers on chest binding (Finney et al., 2024; Jarrett et al., 2018; Julian et al., 2021; Kim et al., 2022; Lee et al., 2019; Pehlivanidis and Anderson, 2024; Patel & Abramowitz, 2020; Peitzmeier et al., 2017, 2021; Reddy-Best et al., 2023; Schultz et al., 2021) and seven on genital tucking (de Nie et al., 2022; Debarbo, 2020; Kidd et al., 2024; Malik et al., 2024; Subedi et al., 2025; Trussler & Carrasquillo, 2020; Turley & Potdar, 2023). There was variability in methodological designs, with quantitative approaches comprising seven cross-sectional studies (Finney et al., 2024; Jarrett et al., 2018; Julian et al., 2021; Malik et al., 2024; Peitzmeier et al., 2017; 2021Kidd et al., 2024), one cohort study (de Nie et al., 2022), and one chart review (Schultz et al., 2021). Additionally, four qualitative studies, three on binding and one on tucking (Lee et al., 2019; Pehlivanidis & Anderson, 2024; Reddy-Best et al., 2023; Subedi et al., 2025), and five case reports (Debarbo, 2020; Kim et al., 2022; Patel & Abramowitz, 2020; Trussler & Carrasquillo, 2020; Turley & Potdar, 2023), three of which reported adverse consequences from tucking (Debarbo, 2020; Trussler & Carrasquillo, 2020; Turley & Potdar, 2023), were included. The total number of participants from included studies was 3,235 individuals, after excluding two studies that shared a similar dataset with the first paper (Peitzmeier et al., 2017), consisting of 392 transfeminine/gender-diverse individuals and 2,843 transmasculine/gender-diverse individuals, of which most studies (*k* = 11) reported or examined the chest binding implications.

Three out of five studies that employed a comparison group focused on chest binding (Finney et al., 2024; Julian et al., 2021; Schultz et al., 2021). One study enrolled participants with chest binding and categorized them based on the usage of commercial binders (Finney et al., 2024); meanwhile, the rest of the studies were comparing between binding and non-binding groups (Julian et al., 2021; Schultz et al., 2021). The age of participants from all case reports ranged from 19 to 29 years. One study focused on youth and adolescent populations with a mean age of 16.4 years (Julian et al., 2021), while, the average age of participants in the remaining quantitative studies ranged between the early to mid-twenties (23 to 26.2 years). The summarized profiles of all included studies on chest binding and genital tucking are shown in Table 2 and Table 3, respectively. The JBI quality checklists were applied based on appropriate study designs and all included studies were rated for methodological quality (see Supplementary Appendix SA1).

**Table 2.** Profiles of all included studies on chest binding.

First author (year), country	Design	Recruitment, Exposure, and Control	Sample size, mean age ± s.d.		Outcomes	Summation of findings and notes
			Case	Control		
Peitzmeier et al. (2017) USA	Cross-sectional	Online survey, binding at the time of the survey or at some point in their lives; 68.1% USA, 13.5% Canada, 7.2% UK, 5.9% other European, 3.8% Oceania; 79.5% transgender, 68.1% male /masculine, 34.2% genderqueer, 33.8% agender; 13.1% received top surgery; binding 10 hours per day and 7 days per week (median); 87.2% using binders; 33.1% using a sports bra	*N* = 1800 Age (median) = 23 (range 18–66)	N/A	28 self-reported health outcomes i.e., back pain, ribs fracture, chest pain, rib or spine changes, shoulder pain, muscle wasting, numbness, overheating, dizziness, fatigue, weakness, acne, itch, scarring, abdominal pain, breast changes, breast tenderness, skin infection, shortness of breath, heartburn, etc.	12.3% very concerned about health effects of binding; Mean 5-point Likert scale of overall mood pre- vs post-binding = 2.1 vs 3.8 (95% CI 1.67–1.79); 97.2% at least one health effect, 76.3% skin/tissue (44.9% itch, 33.9% breast tenderness, 33.8% acne), 74.0% pain (53.8% back, 48.8% chest), 61.7% generalized (53.5% overheating, 27.2% fatigue), 50.7% respiratory (46.6% shortness of breath, 17.2% cough), 46.8% MSK (40.3% bad posture, 11.6% rib or spine changes, 2.8% rib fractures), 41.0% neurological (27.8% lightheadedness or dizziness, 19.1% headache), 17.7% gastrointestinal
Julian et al. (2021), USA	Cross-sectional	Binding vs non-binding; in binding group: 56.4% for 1–4 years, 58.4% everyday, 61.2% 8–16 hours per day, 95.7% learned from online, 90.1% interested in top surgery	*N* = 608 Age= 16.5 ± 2.7	*N* = 76 Age= 15.9 ± 2.9	Self-reported physical impacts, the Chest Dysphoria Scale, life satisfaction subscale of the Gender Congruence & Life Satisfaction scale	80.4% back/chest pain, 67.6% shortness of breath, 60.7% overheating, 59.7% bad posture, 45.1% acne, 33.2% itching, 16.8% rashes, 5.9% other, 4.1% none; no data of physical impacts in the control group; CASE vs CONTROL: mental health service use 41.1% vs 30.3%, medical care use 24.7% vs 15.8%, chest dysphoria scale score 29.0 vs 26.3 (*p* < 0.001), life satisfaction 3.0 vs 2.9; greater chest dysphoria reported lower life satisfaction (*p* < 0.001)
Finney et al. (2024), USA	Cross-sectional	Commercial binder vs non- commercial binder; including 197 transgender and non-binary individuals practicing chest binding (mean age ± s.d. = 26.2 ± 6.6)	*N* = 157 Age= 25.9 ± 6.6	*N* = 40 Age= 27.3 ± 6.4	Positive change scales: self-perception, mental health, public comfort Negative change scales: pain, muscle, skin, and bone changes, and generalized symptoms	Positive change scale scores were significantly higher in case, +0.44 (0.12–0.76, p = 0.008)^b^, and across all subscales but not in negative change scale scores; using a binder for > 10 hours per day was not associated with positive changes, but was associated with higher ratings of negative changes, regardless of the binder category
Jarrett et al. (2018) USA^a^	Cross-sectional	Online survey in binding samples; 57.2% bind every day; binding lifetime duration − 135.9 weeks; 82.9% transgender and 70.7% masculine	*N* = 1273 Age= 25.6 ± 7.5	N/A	Self-reported negative physical symptoms caused by binding, symptom severity, and healthcare management	88.9% any negative symptom; Each domain with ≥ 1 symptom: 77.7% skin/tissue, 74.8% pain, 63.9% generalized, 51.7% respiratory, 48.5% MSK, 41.6% neurological, 18.5% gastrointestinal; 14.8% sought care; 39.1% pain score ≥ 7; 21.0% daily activities limited; HCP issue: 17.7% not important to discuss; 43.7% discomfort to initiate conversion; 53.2% discomfort for chest examination; 43.7% HCP not aware of binding; 6.2% addressed negatively
Peitzmeier et al. (2021) USA^a^	Cross-sectional	Online survey in binding samples; 87% using commercial binders; 30% and 56% bind by age 18 and 21	*N* = 1800 Age= 58% were 18-24	N/A	27 self-reported health outcomes (similar to 01 study except for breast tenderness)	Cumulative exposure as binding-years = intensity (hours per day) × frequency (days per week) × duration (years spent binding); 50% of 10-year prevalence was achieved in < 1 binding-year; pain intensity raised overtime
Schultz et al. (2021), USA	Chart review	Patients underwent top surgery with a recorded history of binding and testosterone use (81.1%); including 74 transmen with mean age = 26.2 (range 15–49); mean binding duration 54.2 months; mean BMI 28.2	*N* = 39 Age = N/A	*N* = 35 Age = N/A	Pathological findings	No malignant lesions were found; a history of binding was not correlated with any benign lesions (*p* = 0.79) or stromal fibrosis (*p* = 0.94); no correlation between any lesions and a history of testosterone use
Patel and Abramowitz (2020), USA	Case report	Comorbid subclinical hypothyroidism, BPD; on weekly testosterone cypionate 120 mg, levothyroxine, aripiprazole, sertraline, quetiapine (dosage not provided); normal estradiol and testosterone at baseline	*N* = 1 Age = 29	N/A	Prolactin level at baseline (m_0_), repeat test (m_2_), 8-week after quetiapine cessation (m_7_), and 4-month after bilateral mastectomy (m_16_)	Prolactin checked due to symptoms associated (daily headache, right-sided blurred vision, and clear discharge of right breast); normal thyroid-stimulating hormone and brain imaging; prolactin level-ng/dL (m_0_:m_2_:m_7_:m_16_): 19.8:40.5:24.6:12.9 (reference 4.0–15.2); unwilling to discontinue chest binding and underwent top surgery
Kim et al. (2022), USA	Case report	Presented by left upper quadrant abdominal pain and needed EGD; comorbid PTSD, BPD, anxiety and panic disorder; on testosterone cypionate, leuprolide, sertraline, zolpidem, ergocalciferol, pantoprazole (dosage not provided); BMI 46.9	*N* = 1 Age = 19	N/A	Oxygen saturation during EGD	Initial EGD operation, 250 mg propofol-induced and SaO_2_ was stable; 4 minutes later, SaO_2_ rapidly decreased to 50% and lost end-tidal respirations; endoscope was removed, additional 200 mg propofol & 20 mg succinylcholine given, and bag-mask ventilated; SaO_2_ recovered to 80% and 100% after 2 and 5 minutes with 100% inspired oxygen; EGD continued; later disclosing wearing a chest binder
Lee et al. (2019), Australia	Qualitative	Convenience sampling of transgender men from Sydney, Australia recruited from social media	*N* = 10 Age (range) = 18-36	N/A	4 themes identified: diversity in roles and meaning negotiating (dis)comfort perceptions of safety interactions with HCP	Thematic analysis; sub-themes: 1.1 distress and authenticity, 1.2 agency and the social world, 2.1 discomfort, 2.2 comfort, 2.3 negotiations, 3.1 community worries, 3.2 mediating public narratives of fear, 3.3 adopting safety measures
Pehlivanidis and Anderson (2024), Australia	Qualitative	Convenience and snowball sampling of transmasculine Australians; semi-structured interview; reflexive thematic analysis	*N* = 15 Age= 26.9 ± 4.6	N/A	4 implication-related themes identified: alleviating distress and improved well-being baseline experiences of negative physical discomfort contextual and environmental complexities of negative experiences binding’s relationship with clothing and dress	Most participants reported some levels of discomfort (pain, irritation, limited breathing capacity) from wearing binding materials, either at the time of wear or after removal; no positive physical effects reported; correct size might not help; influenced by weather, exercise, work, and intimacy; mixed implications on clothing: freedom of expression through clothing but contradicted by the certain outfit of commercial binder
Reddy-best et al. (2023), USA	Qualitative	Online survey with closed and open questionnaires	*N* = 61 Age = 25 (range 18–37)	N/A	Positively influence their self-perception and experiences; physical health concerns; physical and emotional comfort	59% practiced to enhance self-perception and experiences, alleviated distress; 44% chose not to practice because of health concern; some reported discomfort for labor works

s.d.: standard deviation; N/A: not applicable; MSK: musculoskeletal; HCP: health care professionals; BPD: bipolar disorder; ADHD: attention-deficit/hyperactivity disorder; GAD: generalized anxiety disorder; BMI: body mass index; aOR: adjusted odds ratio; EGD: oesophagogastroduodenoscopy; PTSD: posttraumatic stress disorder; SaO_2_: oxygen saturation.

^a^Secondary analysis from the data of Peitzmeier et al. (2017); ^b^Adjusted mean difference and 95% confidence interval.

**Table 3.** Profiles of all included studies on genital tucking.

First author (year), country	Design	Recruitment, Exposure, and Control	Sample size, mean age ± s.d.		Outcomes	Summation of findings and notes
			Case	Control		
Malik et al. (2024), USA	Cross-sectional	Self-reported ever tucked vs never tucked	*N* = 59 Age = 36	*N* = 20 Age = 33	Self-reported health problems	40.7% no problems, 27.1% itch, 20.3% rash, 17.0% pain in testicles, 13.6% pain in penis, 11.9% skin infection, 10.2% urinary tract or bladder infection, 8.5% skin changes, 6.8% problems ejaculating, 6.8% problems urinating: bruised hip (*n* = 1), blood clot in testicles (*n* = 1) and blood clot in urine (*n* = 1)
Kidd et al. (2024), USA	Cross-sectional	Online survey, using closed and open questionnaires; 79% reported tucking; 35% daily, 17% a couple of days/week, 13% a couple of days/month or every few months, and 14% a couple of days/year	*N* = 98 Age (range) = 18–70	N/A	Self-reported complications	65% experienced at least 1 side effect; 32% gonad pain; 10% sought medical care for their side effects; risk of side effects increased with cumulative daily duration of tucking *χ*^2^ (5, *n* = 75) = 11.27; *P* = 0.046.
de Nie et al. (2022), Netherlands	Cohort	Categorized as (1) never, (2) sometimes (1-8x/month), 3) often (>8x/month); median: frequency 5 days per month, duration 6.5 hours per day last binding 12 days ago; total mean age ± s.d. = 24.1 ± 5.8)	*N* = 26 16 some-times 10 often Age = N/A	*N* = 87 (from never group) Age = N/A	Poor semen parameters, logistic regression: never vs sometimes and never vs often (or always in wearing tight undergarments) univariate & multivariate	Multivariate (adjusted age, BMI, smoking, alcohol, cannabis, history of hormone, depression, anxiety, urological problems): statistically significant only for sperm concentration <15x10^6^/mL (aOR 5.48 95%CI 1.18–25.59) akin to wearing tight undergarments, which additionally reached statistical significant in progressive motility <32% in never vs always
Debarbo (2020), Philippines	Case report	Transgender woman; frequency 2–4 times per week; cyproterone acetate 2 mg/day & ethinyl estradiol 3 mcg/day for 6 years	*N* = 1 Age = 24	N/A	Right testicular torsion with a history of 10 hours right scrotal pain	Previously tolerable discomfort and slight pain; participant first decided on bilateral orchiectomy but changed to right unilateral after informing of risks (infertility); pronouns ‘he/his’ were used
Trussler and Carrasquillo (2020), USA	Case report	Comorbid ADHD, GAD, and depression; on bupropion 450 mg/day & lisdexamfetamine 40 mg/day for 7 years; previous daily use of cannabis; normal hormonal status at baseline	*N* = 1 Age = 27	N/A	Karyotyping and genetic testing during tucking; semen parameters during tucking (d_0_) and follow-up at 5-week (w_5_) & 3-month (m_3_)	46 XY, Y-chromosome microdeletion not detected; parameters (d_0_:m_3_): volume-mL (5.7:4.3), pH (7.2: 7.2), %viability (15.4:N/A), total count-x10^6^/mL (0.0:182.8), whole sperm-wet prep (77:N/A), total motile-x10^6^/mL (0.0:62.2), %morphology normal (0.0:1.0), number of sperm vials cryopreserved (N/A:8); w_5_ parameters not provided; unable to cease cannabis use and worse dysphoria after tucking cessation; re-tucking after m_3_
Turley and Potdar (2023), UK	Case report	Referral of a patient for gamete cryopreservation; tucking profiles and hormonal status not provided	*N* = 1 Age between 20–30	N/A	Semen parameters during tucking (m_0_) and follow-up at 2 & 3 months after tucking cessation (m_2_, m_3_)	Parameters (m_0_:m_2_:m_3_): volume-mL (3.2:2.4:3.0), pH (8:8:8), concentration-x10^6^/mL (<1:72:116), %progressive motility (<1:73:47), %morphology normal (<1:9:8), nomenclature: m_0_ oligoastheno- teratozoospermia, m_2_ & m_3_ teratozoospermia, number of sperm vials cryopreserved (1:4:5)
Subedi et al. (2024), USA	Qualitative	Online survey with open ended questions	*N* = 99 Age = 32.35 (range 20–72)	N/A	Self-reported information about gender-affirming care and tucking practices	Dysphoria as a motivation for tucking (alignment with internal sense of gender, desire to pass when wearing tight clothes, for safety reason); the overlooked health implications of tucking (discomfort discussing tucking with healthcare providers, importance of provider knowledge and trust-building practices)

s.d.: standard deviation; BMI: body mass index; N/A: not applicable.

Our review synthesized studies on the implications of chest binding and genital tucking using both quantitative and qualitative designs. The included qualitative research aimed to provide comprehensive insights into the meanings, motivations, implications, and practices of binding and tucking, as well as how information about these practices was disseminated within the community. The developed themes suggested both positive implications, aligning with the participants’ motivations, and negative consequences. Among all quantitative studies and case reports, most reported adverse consequences of these gender affirming practices or investigated self-reported outcomes negatively affected, such as health complications and issues associated with seeking treatment (Jarrett et al., 2018; Julian et al., 2021; Kidd et al., 2024; Lee et al., 2019; Malik et al., 2024; Pehlivanidis & Anderson, 2024; Peitzmeier et al., 2017, 2021; Reddy-Best et al., 2023; Subedi et al., 2025).

However, two studies explored both negative and positive implications, comparing binding and non-binding (Julian et al., 2021) or commercial and noncommercial binders (Finney et al., 2024). They used objective approaches like scoring on chest dysphoria, life satisfaction, or changes in both positive and negative aspects. Additionally, three studies on tucking (de Nie et al., 2022; Trussler & Carrasquillo, 2020; Turley & Potdar, 2023) and one study on binding (Schultz et al., 2021) explored health parameters, evidenced by semen analysis and pathological findings.

## Discussion

### Methodological quality of included studies

Most studies demonstrated fair to good methodological designs but were limited by the absence of long-term follow-up. Sample sizes varied considerably, with the largest study comprising up to 1,800 participants. A significant portion of the included studies were case reports, which inherently lack scientific rigor compared to other study designs. The study populations were diverse, encompassing not only individuals with gender dysphoria but also other sexually and gender-diverse participants. Additionally, the predominant reliance on self-reported surveys might introduce potential recall bias, affecting the reliability of the findings.

### Chest binding

For chest binding, a significant number of negative health implications have been reported, with rates as high as 97.2%, particularly concerning the localized areas exposed to binders, such as dermatologic consequences and pain (Jarrett et al., 2018; Julian et al., 2021; Peitzmeier et al., 2017). Recommendations for addressing dermatologic issues have been proposed (Huang et al., 2022), suggesting both transgender individuals and healthcare professionals have roles in preventing these reactions such as selecting the proper size of binder and considering appropriate medications. Although specific recommendations for chest binding pain have not yet been established, selecting the proper size of binder may also be helpful, especially for acute pain after the first chest binding practice.

Additionally, a multimodal approach for chronic musculoskeletal pains should be considered (El-Tallawy et al., 2021), as they are potentially a cause of chest binding pain, which begins within a year after the first binding (acute pain) and increases its intensity over time (chronic pain), as reported in one included study (Peitzmeier et al., 2021). Meanwhile, more serious localized implications such as breast malignant lesions were reported to have no association with chest binding (Schultz et al., 2021); however, the sample size was only 39.

All studies mentioned above were primarily conducted in transgender individuals from Western countries, where breast density may differ from that of individuals in other regions and ethnicities (McCarthy et al., 2016). Individuals with larger breasts may suffer more from chest binding complications, and the prevalence of such implications may be lower among other ethnicities (Pehlivanidis & Anderson, 2025). Additionally, as suggested by one included qualitative study from Australia (Pehlivanidis & Anderson, 2024), differences in environmental contexts, including factors such as humidity, temperature, and pollution (Reinikainen & Jaakkola, 2003; Zhang et al., 2023), may contribute to variations in the prevalence of dermatologic complications. This highlights the necessity for further studies conducted in non-Western regions where these environmental factors differ.

One case report noted symptomatic hyperprolactinemia potentially due to nipple stimulation caused by chest binding, which improved after bilateral mastectomy (Patel & Abramowitz, 2020). The authors hypothesized that this consequence occurred due to a binder that was too tight, highlighting, once again, the importance of proper size selection. While this is the only case report indicating this condition included in our review, some similar reports have been excluded because of their non-peer-reviewed status and insufficient data such as abstracts. These forms of publications are common for case reports, raising caution about underestimating negative implications informed by case reports, especially in reviews with restricted eligibility criteria.

In addition to the general negative implications of chest binding, one case report highlighted immediate and life-threatening complications during anesthetic procedures, specifically oxygen desaturation, when chest binding was concurrently practiced (Kim et al., 2022). It’s imperative to ensure a correct understanding of chest binding practices, but few studies have examined this issue. Some qualitative studies in our review provided insights (Lee et al., 2019; Pehlivanidis & Anderson, 2024; Reddy-Best et al., 2023); however, they were conducted within specific sociocultural contexts and may have limited generalizability, particularly to individuals with impaired decision-making capacity such as those with severe mental illnesses (Marcó-García et al., 2024). Two aforementioned case reports (Kim et al., 2022; Patel & Abramowitz, 2020) involved individuals with concurrent psychiatric disorders who experienced severe complications from chest binding, highlighting the vulnerability of this population. Therefore, education about chest binding practices and their potential risks should be carefully tailored and monitored for individuals with psychiatric comorbidities.

Much research has reported mental health improvements following hormonal or surgical gender affirmations (Nimitpanya et al., 2022; Park et al., 2022; Pliensak et al., 2023). These affirming practices have been recognized as standard gender-affirming practices, leading to a substantial amount of research in this field. In contrast, non-medical affirming practices such as binding and tucking have received less academic attention concerning their benefits, despite being prevalent practices, high accessibility, and potential for reducing dysphoria and improving mental health status.

Two studies provided evidence of positive changes in self-perception and mental health associated with chest binding (Finney et al., 2024; Julian et al., 2021). This included enhancements in life satisfaction and public comfort, as well as reductions in dysphoria, a primary source of distress experienced by transgender individuals seeking affirmation. Additionally, the other two qualitative studies confirmed the advantages of distress alleviation through chest binding (Pehlivanidis & Anderson, 2024; Reddy-Best et al., 2023). However, it is still challenging to balance the mental benefits against the adverse physical complications. Proper use of chest binding is ultimately of utmost important.

### Genital tucking

For genital tucking, about half of the included studies (*k* = 3) on negative implications were case reports, which included a report of testicular torsion (Debarbo, 2020), and two papers on poorer semen quality (Trussler & Carrasquillo, 2020; Turley & Potdar, 2023). Various semen parameters indicating poorer semen quality were affirmed by one cohort study (de Nie et al., 2022). Despite clear anatomical and pathophysiological explanations of these two complications of genital tucking, they also raise concerns about fertility issues, and transgender individuals should be aware of these risks before practicing genital tucking. According to the World Professional Association for Transgender Health (WPATH) Standards of Care Version 8 for those considering gender-affirming treatments, healthcare professionals should provide transgender individuals with health education on genital tucking benefit and risk (Coleman et al., 2022), while considering potential barriers to fertility services such as resource constraints and religious restrictions when discussing reproductive options (Muhsin et al., 2024; Nadgauda & Butts, 2024).

Similar to chest binding, Malik et al reported dermatologic reactions and testicular pain as the two most frequent complications of genital tucking (Malik et al., 2024). Approximately one-third of individuals who tucked reported of testicular pain (Kidd et al., 2024). However, the reported numbers of these anatomical area-related complications were much smaller (17.0%–32.0%) than those in chest binding (74.0%–76.3%) (Kidd et al., 2024; Malik et al., 2024). Both hormonal and sex/gender differences influence pain perception (Archey et al., 2019), which creates particular complexity for transgender individuals given gender-related factors such hormonal affirmation status, sex assigned at birth, and diverse gender identities and expressions that may affect pain experiences differently.

Additionally, hormonal affirmation for transgender women reduces the testicular volume (Moustakli & Tsonis, 2023), potentially resulting in a lower prevalence of reported pain, whereas transgender men do not experience a similar reduction in breast fat nor fibrous content from their testosterone hormonal affirmation (Chaum et al., 2023; Heng et al., 2024). This difference suggests that medical gender affirmation may influence the implications of non-medical gender affirming practices, as individuals with larger breast volumes might face a higher risk of negative implications from chest binding, as discussed earlier.

One qualitative study described the motivation for tucking as reducing dysphoria, potentially highlighting its benefits for distress alleviation (Subedi et al., 2025). Meanwhile, none of the included quantitative studies has yet investigated the positive implications of genital tucking as primary objectives. However, two studies observed increased suffering from dysphoria after discontinuation of genital tucking (Trussler & Carrasquillo, 2020; Turley & Potdar, 2023), which could imply positive implications for reducing dysphoria, similar to what has been observed with chest binding.

Despite limited evidence supporting the positive implications of these practices in our review, one scoping review suggested that some unpublished qualitative studies report benefits in alleviating chest dysphoria (Pehlivanidis & Anderson, 2025). Due to the methodologies of the existing studies, the causal benefits of these practices require further investigation. A significant number of transgender individuals continue to engage in genital tucking and chest binding, likely influenced by perceived advantages and recommendations from others within transgender communities (Boskey et al., 2023; Malik et al., 2024). However, advice from transgender peers may not always prioritize health concerns, as evidenced by and subsequently resulting in only half of the individuals utilizing these affirming practices being concerned about health effects (Peitzmeier et al., 2017). This underscores the importance of healthcare professionals and the need for comprehensive education to prevent serious complications, particularly among aforementioned vulnerable populations.

Beyond symptom relief, binding and tucking may play important roles in identity management, daily functioning, and social participation. For some, these practices enable safer navigation of public spaces, employment, and relationships, reducing exposure to discrimination or violence. However, they can also create psychosocial strain if concealment is constant or if fear of discovery persists. Understanding these psychosocial dimensions is crucial for tailoring supportive interventions.

### Healthcare interactions and system challenges

Some studies have indicated that many healthcare providers are not familiar with the practices of chest binding and genital tucking or their health implications, resulting in possible care and guidance gaps (Jarrett et al., 2018; Lee et al., 2019; Subedi et al., 2025). Additionally, stigma and discrimination fears prevent individuals from seeking needed medical care, increasing health risks. In addition to promoting awareness, the attitudes of healthcare providers toward transgender individuals play a crucial role in fostering an open environment that encourages transgender individuals to discuss their gender-affirming practices (Wiwattarangkul & Wainipitapong, 2023). Healthcare providers should actively inquire about non-medical gender affirming practices, as failure to do so can lead to severe complications during medical procedures (Kim, 2022), underscoring the critical importance of comprehensive pre-procedural assessments. Healthcare providers may also recommend limiting the duration of continuous genital tucking and chest binding sessions and incorporating regular breaks to prevent complications. This approach aligns with harm reduction principles, recognizing that rather than discouraging these practices entirely, the focus should be on minimizing physical risks while preserving their mental health benefits through education, proper techniques, and clinical support.

### Limitations

The methodologies used across the studies within this systematic review, while being comprehensive, also reveal inherent limitations. A significant constraint is the reliance on self-reported data across many quantitative studies. Such data are prone to biases and inaccuracies from self-assessment, particularly concerning the subjective nature of both physical and psychological symptoms. While qualitative research and case reports cannot represent the overall population, they do complement quantitative findings and offer deeper insights into personal experiences and social contexts surrounding the matter.

Our review was limited by the predominant focus on physical health outcomes in the existing literature, with insufficient research examining mental health benefits, holistic care approaches, or outcomes specific to other gender-diverse individuals such as non-binary, highlighting the need for more comprehensive and inclusive research in this field.

We included only peer-reviewed published works in our review, which may have led to the exclusion of some dissertations or unpublished resources. Nevertheless, our findings are consistent with those of a scoping review that included articles from these two excluded sources. Additionally, PubMed was not included as a separate database in our search strategy, although MEDLINE indexing captures a substantial portion of PubMed content. This choice may have led to the omission of relevant studies that are only indexed in PubMed.

Another limitation is that we included only studies published in English. This may have excluded relevant research in other languages, particularly from non-Western countries, and potentially limited our understanding of cultural variations in binding and tucking practices.

Despite these limitations, this review provides a comprehensive approach, drawing from a range of studies to highlight the significant health implications—both positive and negative—associated with these gender-affirming practices and important areas for future investigation.

### Future research

Future research could prioritize longitudinal studies to better understand the long-term effects of chest binding and genital tucking. Such studies are crucial for examining how these practices impact health over time and whether any negative effects can be reversed. Additionally, there is a notable gap in research focused on improving these practices. Investigating safer and more comfortable methods could offer improved alternatives. Moreover, future research should specifically examine the comparative safety profiles of different chest binding methods, including purpose-made binders, sports bras, and taping techniques. Such research would enable more nuanced, method-specific guidance for transgender individuals choosing between various binding techniques and help healthcare providers offer tailored advice based on individual circumstances and preferences. Finally, and most critically, given the lack of familiarity among healthcare providers with these practices, research studies aimed at promoting education and awareness are crucial for more informed healthcare responses, building the foundation for a supportive and understanding healthcare environment.

## Conclusion

Non-medical gender affirming practices such as chest binding and genital tucking are simple practices with complicated implications. Our review reveals a consistent pattern of tradeoffs between psychological benefits and physical risks. While these practices provide significant mental health benefits for transgender individuals, they should be undertaken with comprehensive awareness of potential adverse effects to support informed decision-making that optimizes both psychological wellbeing and physical safety. The review also identified a significant gap in healthcare professional awareness and education regarding these practices, which affects the quality of care provided to transgender individuals. Addressing this knowledge gap through targeted education and clinical guidelines is essential for supporting safe practice and improving healthcare outcomes for this population.

## Supplementary Material

PRISMA_2020_checklist 280625.docx

Supplementary Appendix 140725.docx
