# Supplementary material for: Non-Medical Gender Affirming Practices Among Transgender Individuals: A Systematic Review on the Health Implications of Chest Binding and Genital Tucking
Source: Int J Sex Health. 2025 Sep 15;38(1):15–29. doi: 10.1080/19317611.2025.2560416 (PMC13094241; doi:10.1080/19317611.2025.2560416)
Supplement: Supplementary Appendix 140725.docx [file WIJS_A_2560416_SM4557.docx]

**Supplementary Appendix SA1 – the Joanna Briggs Institute Quality Appraisal Checklist results**

Peitzmeier S et al. (2017) Health impact of chest binding among transgender adults: a community-engaged, cross-
 sectional study^1^

| **The Critical Appraisal Checklist for analytical cross-sectional study** | | | | |
| --- | --- | --- | --- | --- |
| **Major Components** | **Response options** | | | |
| 1. Were the criteria for inclusion in the sample clearly defined? | **Yes** | No | Unclear | Not applicable |
| 2. Were the study subjects and the setting described in detail? | **Yes** | No | Unclear | Not applicable |
| 3. Was the exposure measured in a valid and reliable way? | Yes | No | **Unclear** | Not applicable |
| 4. Were objective, standard criteria used for measurement of the condition? | Yes | No | **Unclear** | Not applicable |
| 5. Were confounding factors identified? | Yes | No | **Unclear** | Not applicable |
| 6. Were strategies to deal with confounding factors stated? | Yes | No | **Unclear** | Not applicable |
| 7. Were the outcomes measured in a valid and reliable way? | Yes | No | **Unclear** | Not applicable |
| 8. Was appropriate statistical analysis used? | **Yes** | No | Unclear | Not applicable |
| **Overall appraisal:** Include [X] Exclude [ ] Seek further info [ ] | | | | |

Julian J. et al. (2020) The Impact of Chest Binding in Transgender and Gender Diverse Youth and Young Adults^2^

| **The Critical Appraisal Checklist for analytical cross-sectional study** | | | | |
| --- | --- | --- | --- | --- |
| **Major Components** | **Response options** | | | |
| 1. Were the criteria for inclusion in the sample clearly defined? | **Yes** | No | Unclear | Not applicable |
| 2. Were the study subjects and the setting described in detail? | **Yes** | No | Unclear | Not applicable |
| 3. Was the exposure measured in a valid and reliable way? | Yes | No | **Unclear** | Not applicable |
| 4. Were objective, standard criteria used for measurement of the condition? | Yes | No | **Unclear** | Not applicable |
| 5. Were confounding factors identified? | Yes | No | **Unclear** | Not applicable |
| 6. Were strategies to deal with confounding factors stated? | Yes | No | **Unclear** | Not applicable |
| 7. Were the outcomes measured in a valid and reliable way? | **Yes** | No | Unclear | Not applicable |
| 8. Was appropriate statistical analysis used? | **Yes** | No | Unclear | Not applicable |
| **Overall appraisal:** Include [X] Exclude [ ] Seek further info [ ] | | | | |

Malik M. et al. (2023) Tucking Practices and Attributed Health Effects in Transfeminine Individuals^3^

| **The Critical Appraisal Checklist for analytical cross-sectional study** | | | | |
| --- | --- | --- | --- | --- |
| **Major Components** | **Response options** | | | |
| 1. Were the criteria for inclusion in the sample clearly defined? | **Yes** | No | Unclear | Not applicable |
| 2. Were the study subjects and the setting described in detail? | **Yes** | No | Unclear | Not applicable |
| 3. Was the exposure measured in a valid and reliable way? | **Yes** | No | Unclear | Not applicable |
| 4. Were objective, standard criteria used for measurement of the condition? | Yes | No | **Unclear** | Not applicable |
| 5. Were confounding factors identified? | Yes | **No** | Unclear | Not applicable |
| 6. Were strategies to deal with confounding factors stated? | Yes | **No** | Unclear | Not applicable |
| 7. Were the outcomes measured in a valid and reliable way? | **Yes** | No | Unclear | Not applicable |
| 8. Was appropriate statistical analysis used? | **Yes** | No | Unclear | Not applicable |
| **Overall appraisal:** Include [X] Exclude [ ] Seek further info [ ] | | | | |

de Nie E. et al. (2022) A cohort study on factors impairing semen quality in transgender women^4^

| **The Critical Appraisal Checklist for cohort study** | | | | |
| --- | --- | --- | --- | --- |
| **Major Components** | **Response options** | | | |
| 1. Were the two groups similar and recruited from the same population? | **Yes** | No | Unclear | Not applicable |
| 2. Were the exposures measured similarly to assign people to both exposed and unexposed   groups? | **Yes** | No | Unclear | Not applicable |
| 3. Was the exposure measured in a valid and reliable way? | Yes | No | **Unclear** | Not applicable |
| 4. Were confounding factors identified? | **Yes** | No | Unclear | Not applicable |
| 5. Were strategies to deal with confounding factors stated? | **Yes** | No | Unclear | Not applicable |
| 6. Were the groups/participants free of the outcome at the start of the study (or at the moment   of exposure)? | Yes | **No** | Unclear | Not applicable |
| 7. Were the outcomes measured in a valid and reliable way? | Yes | No | Unclear | Not applicable |
| 8. Was the follow up time reported and sufficient to be long enough for outcomes to occur? | Yes | No | **Unclear** | Not applicable |
| 9. Was follow up complete, and if not, were the reasons to loss to follow up described and   explored? | Yes | No | Unclear | **Not applicable** |
| 10. Were strategies to address incomplete follow up utilized? | Yes | No | Unclear | **Not applicable** |
| 11. Was appropriate statistical analysis used? | Yes | No | **Unclear** | Not applicable |
| **Overall appraisal:** Include [X] Exclude [ ] Seek further info [ ] | | | | |

Finney N. et al. (2023) Physical and Mental Changes Reported by Transgender and Non-Binary Users of Commercial and
 Non-Commercial Chest Binders: A Community-Informed Cross-Sectional Observational Study^5^

| **The Critical Appraisal Checklist for analytical cross-sectional study** | | | | |
| --- | --- | --- | --- | --- |
| **Major Components** | **Response options** | | | |
| 1. Were the criteria for inclusion in the sample clearly defined? | **Yes** | No | Unclear | Not applicable |
| 2. Were the study subjects and the setting described in detail? | **Yes** | No | Unclear | Not applicable |
| 3. Was the exposure measured in a valid and reliable way? | **Yes** | No | Unclear | Not applicable |
| 4. Were objective, standard criteria used for measurement of the condition? | **Yes** | No | Unclear | Not applicable |
| 5. Were confounding factors identified? | Yes | **No** | Unclear | Not applicable |
| 6. Were strategies to deal with confounding factors stated? | Yes | **No** | Unclear | Not applicable |
| 7. Were the outcomes measured in a valid and reliable way? | **Yes** | No | Unclear | Not applicable |
| 8. Was appropriate statistical analysis used? | **Yes** | No | Unclear | Not applicable |
| **Overall appraisal:** Include [X] Exclude [ ] Seek further info [ ] | | | | |

Jarrett B. et al. (2018) Chest Binding and Care Seeking Among Transmasculine Adults: A Cross-Sectional Study^6^

| **The Critical Appraisal Checklist for analytical cross-sectional study** | | | | |
| --- | --- | --- | --- | --- |
| **Major Components** | **Response options** | | | |
| 1. Were the criteria for inclusion in the sample clearly defined? | **Yes** | No | Unclear | Not applicable |
| 2. Were the study subjects and the setting described in detail? | **Yes** | No | Unclear | Not applicable |
| 3. Was the exposure measured in a valid and reliable way? | Yes | **No** | Unclear | Not applicable |
| 4. Were objective, standard criteria used for measurement of the condition? | **Yes** | No | Unclear | Not applicable |
| 5. Were confounding factors identified? | Yes | No | **Unclear** | Not applicable |
| 6. Were strategies to deal with confounding factors stated? | Yes | No | **Unclear** | Not applicable |
| 7. Were the outcomes measured in a valid and reliable way? | **Yes** | No | Unclear | Not applicable |
| 8. Was appropriate statistical analysis used? | **Yes** | No | Unclear | Not applicable |
| **Overall appraisal:** Include [X] Exclude [ ] Seek further info [ ] | | | | |

Peitzmeier S. et al. (2021) Time to First Onset of Chest Binding–Related Symptoms in Transgender Youth^7^

| **The Critical Appraisal Checklist for analytical cross-sectional study** | | | | |
| --- | --- | --- | --- | --- |
| **Major Components** | **Response options** | | | |
| 1. Were the criteria for inclusion in the sample clearly defined? | **Yes** | No | Unclear | Not applicable |
| 2. Were the study subjects and the setting described in detail? | **Yes** | No | Unclear | Not applicable |
| 3. Was the exposure measured in a valid and reliable way? | Yes | **No** | Unclear | Not applicable |
| 4. Were objective, standard criteria used for measurement of the condition? | Yes | No | **Unclear** | Not applicable |
| 5. Were confounding factors identified? | Yes | No | **Unclear** | Not applicable |
| 6. Were strategies to deal with confounding factors stated? | Yes | No | **Unclear** | Not applicable |
| 7. Were the outcomes measured in a valid and reliable way? | Yes | **No** | Unclear | Not applicable |
| 8. Was appropriate statistical analysis used? | **Yes** | No | Unclear | Not applicable |
| **Overall appraisal:** Include [X] Exclude [ ] Seek further info [ ] | | | | |

Schultz J. et al. (2021) Pathological Evaluation of Breast Specimens in Transgender Chest Masculinization: Incidental
 Findings and Effect of Prior Chest Binding and Androgen Therapy in 74 Consecutive Patients^8^

| **The Critical Appraisal Checklist for analytical cross-sectional study (adapted for chart review design)** | | | | |
| --- | --- | --- | --- | --- |
| **Major Components** | **Response options** | | | |
| 1. Were the criteria for inclusion in the sample clearly defined? | **Yes** | No | Unclear | Not applicable |
| 2. Were the study subjects and the setting described in detail? | **Yes** | No | Unclear | Not applicable |
| 3. Was the exposure measured in a valid and reliable way? | Yes | No | **Unclear** | Not applicable |
| 4. Were objective, standard criteria used for measurement of the condition? | Yes | No | **Unclear** | Not applicable |
| 5. Were confounding factors identified? | **Yes** | No | Unclear | Not applicable |
| 6. Were strategies to deal with confounding factors stated? | **Yes** | No | Unclear | Not applicable |
| 7. Were the outcomes measured in a valid and reliable way? | **Yes** | No | Unclear | Not applicable |
| 8. Was appropriate statistical analysis used? | **Yes** | No | Unclear | Not applicable |
| **Overall appraisal:** Include [X] Exclude [ ] Seek further info [ ] | | | | |

Patel S. & Abramowitz J. (2019) Hyperprolactinemia in a transgender male^9^

| **The Critical Appraisal Checklist for case reports** | | | | |
| --- | --- | --- | --- | --- |
| **Major Components** | **Response options** | | | |
| 1. Were patient’s demographic characteristics clearly described? | Yes | No | **Unclear** | Not applicable |
| 2. Was the patient’s history clearly described and presented as a timeline? | Yes | No | **Unclear** | Not applicable |
| 3. Was the current clinical condition of the patient on presentation clearly described? | **Yes** | No | Unclear | Not applicable |
| 4. Were diagnostic tests or assessment methods and the results clearly described? | **Yes** | No | Unclear | Not applicable |
| 5. Was the intervention(s) or treatment procedure(s) clearly described? | **Yes** | No | Unclear | Not applicable |
| 6. Was the post-intervention clinical condition clearly described? | **Yes** | No | Unclear | Not applicable |
| 7. Were adverse events (harms) or unanticipated events identified and described? | Yes | No | Unclear | **Not applicable** |
| 8. Does the case report provide takeaway lessons? | **Yes** | No | Unclear | Not applicable |
| **Overall appraisal:** Include [X] Exclude [ ] Seek further info [ ] | | | | |

Kim E. et al. (2022) Oxygen desaturation in a transgender man: initial concerns and recommendations regarding the 
 practice of chest binding: a case report^10^

| **The Critical Appraisal Checklist for case reports** | | | | |
| --- | --- | --- | --- | --- |
| **Major Components** | **Response options** | | | |
| 1. Were patient’s demographic characteristics clearly described? | **Yes** | No | Unclear | Not applicable |
| 2. Was the patient’s history clearly described and presented as a timeline? | **Yes** | No | Unclear | Not applicable |
| 3. Was the current clinical condition of the patient on presentation clearly described? | **Yes** | No | Unclear | Not applicable |
| 4. Were diagnostic tests or assessment methods and the results clearly described? | **Yes** | No | Unclear | Not applicable |
| 5. Was the intervention(s) or treatment procedure(s) clearly described? | Yes | No | **Unclear** | Not applicable |
| 6. Was the post-intervention clinical condition clearly described? | **Yes** | No | Unclear | Not applicable |
| 7. Were adverse events (harms) or unanticipated events identified and described? | **Yes** | No | Unclear | Not applicable |
| 8. Does the case report provide takeaway lessons? | **Yes** | No | Unclear | Not applicable |
| **Overall appraisal:** Include [X] Exclude [ ] Seek further info [ ] | | | | |

Lee A. et al. (2019) The binding practices of transgender and gender-diverse adults in Sydney, Australia^11^

| **The Critical Appraisal Checklist for qualitative study** | | | | |
| --- | --- | --- | --- | --- |
| **Major Components** | **Response options** | | | |
| 1. Is there congruity between the stated philosophical perspective and the research   methodology? | **Yes** | No | Unclear | Not applicable |
| 2. Is there congruity between the research methodology and the research question or objectives? | **Yes** | No | Unclear | Not applicable |
| 3. Is there congruity between the research methodology and the methods used to collect data? | **Yes** | No | Unclear | Not applicable |
| 4. Is there congruity between the research methodology and the representation and analysis of   data? | **Yes** | No | Unclear | Not applicable |
| 5. Is there congruity between the research methodology and the interpretation of results? | **Yes** | No | Unclear | Not applicable |
| 6. Is there a statement locating the researcher culturally or theoretically? | **Yes** | No | Unclear | Not applicable |
| 7. Is the influence of the researcher on the research, and vice-versa, addressed? | Yes | No | **Unclear** | Not applicable |
| 8. Are participants, and their voices, adequately represented? | **Yes** | No | Unclear | Not applicable |
| 9. Is the research ethical according to current criteria or is there evidence of ethical approval by an   appropriate body? | **Yes** | No | Unclear | Not applicable |
| 10. Do the conclusions drawn in the research report flow from the analysis, or interpretation, of   the data? | **Yes** | No | Unclear | Not applicable |
| **Overall appraisal:** Include [X] Exclude [ ] Seek further info [ ] | | | | |

Debarbo C et al. (2019) Rare cause of testicular torsion in a transwoman: A case report^12^

| **The Critical Appraisal Checklist for case reports** | | | | |
| --- | --- | --- | --- | --- |
| **Major Components** | **Response options** | | | |
| 1. Were patient’s demographic characteristics clearly described? | **Yes** | No | Unclear | Not applicable |
| 2. Was the patient’s history clearly described and presented as a timeline? | **Yes** | No | Unclear | Not applicable |
| 3. Was the current clinical condition of the patient on presentation clearly described? | **Yes** | No | Unclear | Not applicable |
| 4. Were diagnostic tests or assessment methods and the results clearly described? | **Yes** | No | Unclear | Not applicable |
| 5. Was the intervention(s) or treatment procedure(s) clearly described? | Yes | No | **Unclear** | Not applicable |
| 6. Was the post-intervention clinical condition clearly described? | Yes | **No** | Unclear | Not applicable |
| 7. Were adverse events (harms) or unanticipated events identified and described? | Yes | No | Unclear | **Not applicable** |
| 8. Does the case report provide takeaway lessons? | **Yes** | No | Unclear | Not applicable |
| **Overall appraisal:** Include [X] Exclude [ ] Seek further info [ ] | | | | |

Trussler J. & Carrasquillo R. (2020) Cryptozoospermia Associated With Genital Tucking Behavior in a Transwoman^13^

| **The Critical Appraisal Checklist for case reports** | | | | |
| --- | --- | --- | --- | --- |
| **Major Components** | **Response options** | | | |
| 1. Were patient’s demographic characteristics clearly described? | **Yes** | No | Unclear | Not applicable |
| 2. Was the patient’s history clearly described and presented as a timeline? | **Yes** | No | Unclear | Not applicable |
| 3. Was the current clinical condition of the patient on presentation clearly described? | Yes | No | **Unclear** | Not applicable |
| 4. Were diagnostic tests or assessment methods and the results clearly described? | **Yes** | No | Unclear | Not applicable |
| 5. Was the intervention(s) or treatment procedure(s) clearly described? | Yes | No | **Unclear** | Not applicable |
| 6. Was the post-intervention clinical condition clearly described? | **Yes** | No | Unclear | Not applicable |
| 7. Were adverse events (harms) or unanticipated events identified and described? | Yes | No | Unclear | **Not applicable** |
| 8. Does the case report provide takeaway lessons? | **Yes** | No | Unclear | Not applicable |
| **Overall appraisal:** Include [X] Exclude [ ] Seek further info [ ] | | | | |

Turley R. & Potdar N. (2023) A Case of Oligoasthenoteratozoospermia Following Genital Tucking: Transgender Fertility
 Preservation^14^

| **The Critical Appraisal Checklist for case reports** | | | | |
| --- | --- | --- | --- | --- |
| **Major Components** | **Response options** | | | |
| 1. Were patient’s demographic characteristics clearly described? | Yes | **No** | Unclear | Not applicable |
| 2. Was the patient’s history clearly described and presented as a timeline? | Yes | No | **Unclear** | Not applicable |
| 3. Was the current clinical condition of the patient on presentation clearly described? | **Yes** | No | Unclear | Not applicable |
| 4. Were diagnostic tests or assessment methods and the results clearly described? | **Yes** | No | Unclear | Not applicable |
| 5. Was the intervention(s) or treatment procedure(s) clearly described? | **Yes** | No | Unclear | Not applicable |
| 6. Was the post-intervention clinical condition clearly described? | **Yes** | No | Unclear | Not applicable |
| 7. Were adverse events (harms) or unanticipated events identified and described? | Yes | No | Unclear | **Not applicable** |
| 8. Does the case report provide takeaway lessons? | **Yes** | No | Unclear | Not applicable |
| **Overall appraisal:** Include [X] Exclude [ ] Seek further info [ ] | | | | |

Kidd N. et al. (2024) Genital Tucking Practices in Transgender and Gender Diverse Patients.^15^

| **The Critical Appraisal Checklist for analytical cross-sectional study** | | | | |
| --- | --- | --- | --- | --- |
| **Major Components** | **Response options** | | | |
| 1. Were the criteria for inclusion in the sample clearly defined? | **Yes** | No | Unclear | Not applicable |
| 2. Were the study subjects and the setting described in detail? | **Yes** | No | Unclear | Not applicable |
| 3. Was the exposure measured in a valid and reliable way? | **Yes** | No | Unclear | Not applicable |
| 4. Were objective, standard criteria used for measurement of the condition? | **Yes** | No | Unclear | Not applicable |
| 5. Were confounding factors identified? | **Yes** | No | Unclear | Not applicable |
| 6. Were strategies to deal with confounding factors stated? | Yes | **No** | Unclear | Not applicable |
| 7. Were the outcomes measured in a valid and reliable way? | **Yes** | No | Unclear | Not applicable |
| 8. Was appropriate statistical analysis used? | **Yes** | No | Unclear | Not applicable |
| **Overall appraisal:** Include [X] Exclude [ ] Seek further info [ ] | | | | |

Subedi S. et al. (2024) “I was largely unguided trying to figure it out on my own”: experiences of genital tucking among transfeminine and gender diverse individuals.

| **The Critical Appraisal Checklist for qualitative study** | | | | |
| --- | --- | --- | --- | --- |
| **Major Components** | **Response options** | | | |
| 1. Is there congruity between the stated philosophical perspective and the research   methodology? | **Yes** | No | Unclear | Not applicable |
| 2. Is there congruity between the research methodology and the research question or objectives? | **Yes** | No | Unclear | Not applicable |
| 3. Is there congruity between the research methodology and the methods used to collect data? | Yes | **No** | Unclear | Not applicable |
| 4. Is there congruity between the research methodology and the representation and analysis of   data? | **Yes** | No | Unclear | Not applicable |
| 5. Is there congruity between the research methodology and the interpretation of results? | **Yes** | No | Unclear | Not applicable |
| 6. Is there a statement locating the researcher culturally or theoretically? | **Yes** | No | Unclear | Not applicable |
| 7. Is the influence of the researcher on the research, and vice-versa, addressed? | Yes | **No** | Unclear | Not applicable |
| 8. Are participants, and their voices, adequately represented? | **Yes** | No | Unclear | Not applicable |
| 9. Is the research ethical according to current criteria or is there evidence of ethical approval by an   appropriate body? | **Yes** | No | Unclear | Not applicable |
| 10. Do the conclusions drawn in the research report flow from the analysis, or interpretation, of   the data? | **Yes** | No | Unclear | Not applicable |
| **Overall appraisal:** Include [X] Exclude [ ] Seek further info [ ] | | | | |

Reddy-best K. et al. (2023) Chest-Binding Practices for Trans and Nonbinary Individuals within Different Spatiotemporalities: Redefining the Meanings of Space, Place, and Time.

| **The Critical Appraisal Checklist for qualitative study** | | | | |
| --- | --- | --- | --- | --- |
| **Major Components** | **Response options** | | | |
| 1. Is there congruity between the stated philosophical perspective and the research   methodology? | **Yes** | No | Unclear | Not applicable |
| 2. Is there congruity between the research methodology and the research question or objectives? | **Yes** | No | Unclear | Not applicable |
| 3. Is there congruity between the research methodology and the methods used to collect data? | Yes | **No** | Unclear | Not applicable |
| 4. Is there congruity between the research methodology and the representation and analysis of   data? | **Yes** | No | Unclear | Not applicable |
| 5. Is there congruity between the research methodology and the interpretation of results? | **Yes** | No | Unclear | Not applicable |
| 6. Is there a statement locating the researcher culturally or theoretically? | **Yes** | No | Unclear | Not applicable |
| 7. Is the influence of the researcher on the research, and vice-versa, addressed? | Yes | **No** | Unclear | Not applicable |
| 8. Are participants, and their voices, adequately represented? | **Yes** | No | Unclear | Not applicable |
| 9. Is the research ethical according to current criteria or is there evidence of ethical approval by an   appropriate body? | **Yes** | No | Unclear | Not applicable |
| 10. Do the conclusions drawn in the research report flow from the analysis, or interpretation, of   the data? | **Yes** | No | Unclear | Not applicable |
| **Overall appraisal:** Include [X] Exclude [ ] Seek further info [ ] | | | | |

Pehlivanidis SG, Anderson JR. A qualitative exploration of the motivations and implications of chest binding practices for transmasculine Australians. International Journal of Transgender Health. 2024 0(0):1–14.

| **The Critical Appraisal Checklist for qualitative study** | | | | |
| --- | --- | --- | --- | --- |
| **Major Components** | **Response options** | | | |
| 1. Is there congruity between the stated philosophical perspective and the research   methodology? | **Yes** | No | Unclear | Not applicable |
| 2. Is there congruity between the research methodology and the research question or objectives? | **Yes** | No | Unclear | Not applicable |
| 3. Is there congruity between the research methodology and the methods used to collect data? | **Yes** | No | Unclear | Not applicable |
| 4. Is there congruity between the research methodology and the representation and analysis of   data? | **Yes** | No | Unclear | Not applicable |
| 5. Is there congruity between the research methodology and the interpretation of results? | **Yes** | No | Unclear | Not applicable |
| 6. Is there a statement locating the researcher culturally or theoretically? | **Yes** | No | Unclear | Not applicable |
| 7. Is the influence of the researcher on the research, and vice-versa, addressed? | **Yes** | No | Unclear | Not applicable |
| 8. Are participants, and their voices, adequately represented? | **Yes** | No | Unclear | Not applicable |
| 9. Is the research ethical according to current criteria or is there evidence of ethical approval by an   appropriate body? | **Yes** | No | Unclear | Not applicable |
| 10. Do the conclusions drawn in the research report flow from the analysis, or interpretation, of   the data? | **Yes** | No | Unclear | Not applicable |
| **Overall appraisal:** Include [X] Exclude [ ] Seek further info [ ] | | | | |

**References**

1. Peitzmeier S, Gardner I, Weinand J, et al. Health impact of chest binding among transgender adults: a community-engaged, cross-sectional study. Cult Health Sex. 2017;19(1):64–75; doi: 10.1080/13691058.2016.1191675.
2. Julian JM, Salvetti B, Held JI, et al. The impact of chest binding in transgender and gender diverse youth and young adults. J Adolesc Health. 2021;68(6):1129–1134; doi: 10.1016/j.jadohealth.2020.09.029.
3. Malik M, Cooney EE, Brevelle J-M, et al. Tucking practices and attributed health effects in transfeminine individuals. Transgend Health. 2024;9(1):92–97; doi: 10.1089/trgh.2022.0064.
4. de Nie I, Asseler J, Meißner A, et al. A cohort study on factors impairing semen quality in transgender women. Am J Obstet Gynecol. 2022;226(3):390.e1-390.e10; doi: 10.1016/j.ajog.2021.10.020.
5. Finney N, Slomoff R, Cervantes B, et al. Physical and mental changes reported by transgender and non-binary users of commercial and non-commercial chest binders: A community-informed cross-sectional observational study. Transgender Health. 2023; doi: 10.1089/trgh.2023.0051.
6. Jarrett BA, Corbet AL, Gardner IH, et al. Chest binding and care seeking among transmasculine adults: A cross-sectional study. Transgend Health. 2018;3(1):170–178; doi: 10.1089/trgh.2018.0017.
7. Peitzmeier SM, Silberholz J, Gardner IH, et al. Time to first onset of chest binding-related symptoms in transgender youth. Pediatrics. 2021;147(3):e20200728; doi: 10.1542/peds.2020-0728.
8. Schultz JJ, Naides AI, Bai D, et al. Pathological evaluation of breast specimens in transgender chest masculinization: Incidental findings and effect of prior chest binding and androgen therapy in 74 consecutive patients. Transgender Health. 2021; doi: 10.1089/trgh.2020.0108.
9. Patel S, Abramowitz J. Hyperprolactinemia in a transgender male. AACE Clin Case Rep. 2020;6(1):e5–e8; doi: 10.4158/ACCR-2019-0272.
10. Kim E, Mukerji S, Debryn D, et al. Oxygen desaturation in a transgender man: Initial concerns and recommendations regarding the practice of chest binding: a case report. J Med Case Rep. 2022;16(1):333; doi: 10.1186/s13256-022-03527-z.
11. Lee A, Simpson P, Haire B. The binding practices of transgender and gender-diverse adults in Sydney, Australia. Cult Health Sex. 2019;21(9):969–984; doi: 10.1080/13691058.2018.1529335.
12. Debarbo CJM. Rare cause of testicular torsion in a transwoman: A case report. Urol Case Rep. 2020;33:101422; doi: 10.1016/j.eucr.2020.101422.
13. Trussler JT, Carrasquillo RJ. Cryptozoospermia associated with genital tucking behavior in a transwoman. Rev Urol. 2020;22(4):170–173.
14. Turley R, Potdar N. A Case of Oligoasthenoteratozoospermia following genital tucking: Transgender fertility preservation. Reprod Sci. 2023;30(7):2248–2251; doi: 10.1007/s43032-023-01168-1.
15. Kidd N, Mark K, Dart M, et al. Genital tucking practices in transgender and gender diverse patients. Ann Fam Med. 2024;22(2):149–153; doi: 10.1370/afm.3076.
16. Subedi S, Kant J, Miranda N, et al. “I was largely unguided trying to figure it out on my own”: Experiences of genital tucking among transfeminine and gender diverse individuals. Int J Transgend Health. 2024;0(0):1–12; doi: 10.1080/26895269.2024.2333533.
17. Reddy-best K, Reilly A, Streck K, et al. Chest-binding practices for trans and nonbinary individuals within different spatiotemporalities: Redefining the meanings of space, place, and time. Fashion Theory. 2023;27:1–28; doi: 10.1080/1362704X.2023.2196761.
18. Pehlivanidis SG, Anderson JR. A qualitative exploration of the motivations and implications of chest binding practices for transmasculine Australians. Int J Transgend Health. 2024;0(0):1–14; doi: 10.1080/26895269.2024.2319792.
